# Independent evolution of a living bridge in the old world army ant lineage

**DOI:** 10.1007/s00114-026-02085-4

**Published:** 2026-03-03

**Authors:** Nobuaki Mizumoto, Kôichi Arimoto, Clement Het Kaliang, Taisuke Kanao

**Affiliations:** 1 Department of Entomology & Plant Pathology, Auburn University, Auburn, AL 36849 USA; 2 Okinawa Institute of Science & Technology Graduate University, Onna-son, Okinawa, 904-0495 Japan; 3 Graduate School of Global Environmental Studies, Kyoto University, Kyoto, 606– 8501 Japan; 4 Sarawak Forestry Corporation, Kuching, Sarawak Malaysia; 5 Faculty of Science, Yamagata University, Yamagata, 990-8560 Japan

**Keywords:** Collective motion, Natural history, Self-organization, Self-assembly, Traffic regulation, Southeast Asia

## Abstract

Living bridges formed by army ants are among the most striking examples of collective behavior in social insects, previously known only from New World *Eciton* species. Here, we report the field observation of living bridge formation in an Old World army ant, *Aenictus glabrinotum*. We artificially created a small gap in the foraging trail, formed on a twig, to disrupt traffic flow. Ants accumulated near the opening, explored the air space, and interlinked their bodies to form a living bridge, allowing traffic to resume. Bridges were maintained while traffic was high but spontaneously disassembled as flow declined, suggesting a similar behavioral process to that of *Eciton* behavior. Although it is limited to a single opportunistic observation, this documentation suggests that bridge-formation may have evolved several times in army ants. By motivating further comparative studies across army ant lineages, this study provides new insight into the diversity of self-assembly in social insects.

**Supplementary Information:**

The online version contains supplementary material available at 10.1007/s00114-026-02085-4.

## Introduction

Collective behavior enables groups of animals to perform tasks that individuals cannot accomplish alone. One striking example is self-assembly, in which individuals physically link their bodies to create dynamic and flexible three-dimensional structures (Anderson et al. 2002; Carlesso and Reid 2023). Such structures provide functional advantages, otherwise impossible for single individuals, such as forming temporary shelters, bridging gaps to facilitate movements, or combining forces to achieve greater strength (Anderson et al. 2002; Carlesso and Reid 2023). Self-assembly is rare across animals and is largely restricted to several derived social Hymenopteran species, e.g., fire ant rafts, honey bee swarms, weaver ant chains, and army ant bivouacs (Carlesso and Reid 2023). Although these are system-specific phenomena, similar ecological pressures may promote the convergent evolution of self-assembly behaviors across unrelated taxa.

Army ants represent a particularly charismatic example of collective behavior, exhibiting a suite of traits known as the “army ant syndrome”, including obligate group foraging and nomadic colony cycles (Schneirla 1971; Gotwald 1995; Kronauer 2020). This syndrome has evolved convergently in two lineages: the New World and the Old World army ants (Borowiec 2019). Among these, the genera *Eciton* (New World) and *Aenictus* (Old World) are especially surface-adapted, typically foraging and migrating on open ground, rather than within leaf litter or subterranean tunnels (Gotwald 1995). Their movements depend on the continuity of the ground surface, potentially disrupted by small gaps (Mizumoto and Reid 2024). The New World army ants, *Eciton*, are well known for their ability to overcome such spatial discontinuities by forming living bridges that span gaps, and the behavioral mechanisms underlying this phenomenon have been well documented (Reid et al. 2015; McCreery et al. 2022). In contrast, behavioral and life history information remain scarce in *Aenictus* species. Unlike *Eciton* ants, *Aenictus* lacks polymorphic workers and forms a simpler collective foraging system (Schneirla 1971; Gotwald 1995; Yamane et al. 2021), yet their surface-adapted lifestyle suggests they might also employ similar bridging behaviors.

**Fig. 1:**
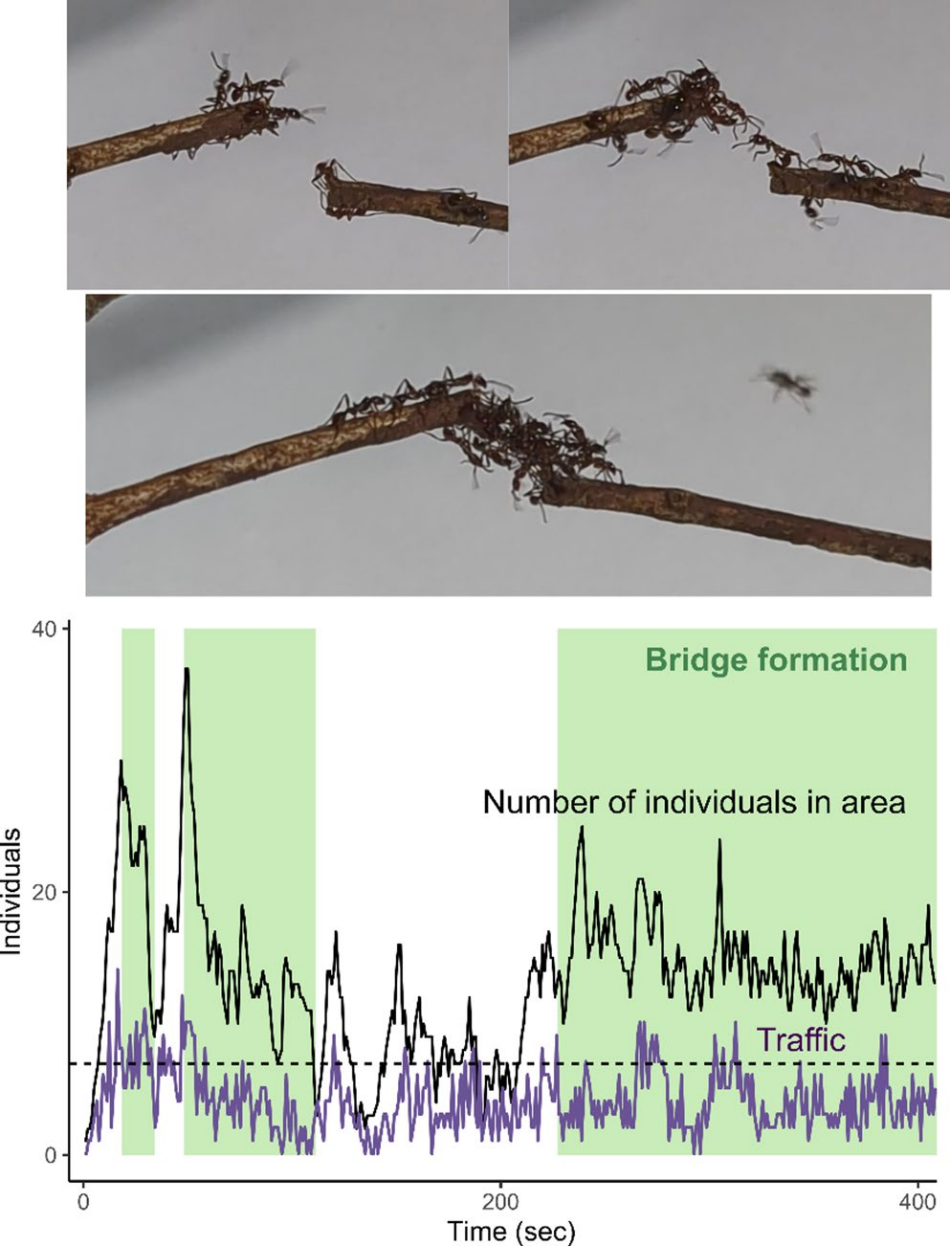
Living bridge formation by *Aenictus glabrinotum*. Time development of the number of individuals around the gap (25 mm) was recorded (see Figure S1 for the scale). Also, traffic was measured as the number of individuals entering or leaving this area per second. The dashed horizontal line indicates the minimum number of ants in the gap area while the bridge was formed

Here, we report observations of living bridges in *A. glabrinotum *(Fig. 1, Video S1). This finding provides rare insight into collective behavior in the Old World army ants and suggests that comparable ecological challenges may have driven similar self-assembly behaviors across independent lineages of army ants.

## Methods

All observations were performed in the Lambir Hills National Park, Miri, Sarawak, Malaysia. Between May 31 and June 12, 2023, we conducted daily field surveys of marching termites (*Hospitalitermes* spp. and *Longipeditermes longipes*) along the ~ 4 km trail (Inoue-Pantu-Main trail), walking the route once or twice per day to search for termite trails. During these surveys, we occasionally encountered foraging or emigration trails of *Aenictus* spp., both during the day and at night. On June 6, while returning to the chalet after observations on *Hospitalitermes lividiceps*, we encountered the tail of an *A. glabrinotum* colony at 10:20 pm local time (4.2023437° N, 114.0302110° E). There are 18 *Aenictus* species in the Lambir Hills National Park (Yamane et al. 2021), and the information about the species-level identity is provided in the Supplementary Text S1. The trail crossed a narrow twig of dead wood on the forest floor, providing a natural opportunity to test whether this species could form a living bridge when the substrate was interrupted.

We cut the twig bridge with scissors and recorded the ant responses using the 4 K video camera (HC-X1500-K, Panasonic) at 30 fps, illuminated by an LED video light (Pixel G1s) (Fig. 1). The resulting gap was approximately 5.3 mm wide, slightly larger than the body length of ants (4.11 ± 0.34 mm, Mean ± S.D.; *n* = 10, measured from the snapshot of the video). Preliminary observations indicated that LED lights at night did not disturb trail activity. Recording lasted for about 400 s, limited by battery capacity and safety considerations for returning from the field site.

To quantify traffic dynamics during bridge formation, we analyzed the video using the open-source event-logging software BORIS (Friard and Gamba 2016). The observation area was defined as a 50 × 50 mm square centered on the twig gap (Fig. S1). We counted the number of ants entering and leaving the area each second and binned the data accordingly. The traffic flow was defined as the total number of individuals entering or leaving the area per second, and the estimated number of ants present was derived from cumulative entry and exit counts over the observation period. These data were used to describe the temporal dynamics of collective bridge formation.

The autocorrelation function of the traffic flow data shows no detectable correlation at lags > 11 s (Fig. S2). Thus, we obtained the total traffic flow and the mean number of ants near the gap for each 11-second bin. We investigated the effects of the mean number of ants near the gap, bridge formation, and interactions on traffic flow using a generalized linear model (GLM) with a Poisson error distribution and a log link function. The likelihood ratio test was used to determine the statistical significance of each explanatory variable (type II test). All analyses were performed using R (R Core Team 2024).

## Results

During the observation period, we observed three bridge formation events and two spontaneous collapses (Fig. 1). After a gap was created, traffic was disrupted, and some ants went back in the other direction, while others stayed near the gap to explore the air by moving their antennae and fore legs (Video S1). Traffic disruptions sometimes led to congestion, with ants accumulating near the opening (Fig. 1). The decision to explore the airspace or turn back seemed to be made individually, rather than one forcing the other. When ants from both sides explored the air and made contact, they antennated each other, then interlocked their bodies to form a living bridge. Once the bridge was established, traffic resumed, and the local ant density around the gap decreased. The bridge persisted as long as ants continued to cross but collapsed spontaneously when traffic declined (Fig. 1).

During bridge formation, the minimum number of ants present in the gap area was seven, indicating that at least seven individuals were required to span the 5.3 mm gap. The number of ants near the gap was positively correlated with traffic flow (GLM; slope, 95%CI 0.039—0.066, χ^2^₁ = 125.4, *P* < 0.001, Fig. S3, Table S1), and the slope of this relationship was significantly steeper when the bridge was present (GLM; interaction, 95%CI: 0.002—0.047 χ^2^_1_ = 4.7, *P* = 0.03, Fig. S3, Table S1).

## Discussion

Old World and New World army ants show many convergent traits, yet also display key differences in their collective behaviors. Both *Aenictus* and *Eciton* are surface-adapted foragers (Gotwald 1995), but *Eciton* species typically form larger and more complex raid networks, while *Aenictus* raids tend to be simpler (Schneirla 1971; Gotwald 1995). Our observations reveal that *Aenictus* ants can also form living bridges. However, bridge formation in *Aenictus* appears more limited, typically spanning only small gaps and often breaking down spontaneously (Fig. 1). This likely reflects the lower overall traffic intensity in *Aenictus* trails (Schneirla and Reyes 1966). Also, as the gap was artificially introduced in this study, it remains unknown whether *Aenictus* forms a living bridge to actively cross the gap or only to repair the existing trail. Repetitive observations on larger-scale trials could further clarify the bridge formation by *Aenictus* ants, confirming our anecdotal observation.

**Fig. 2:**
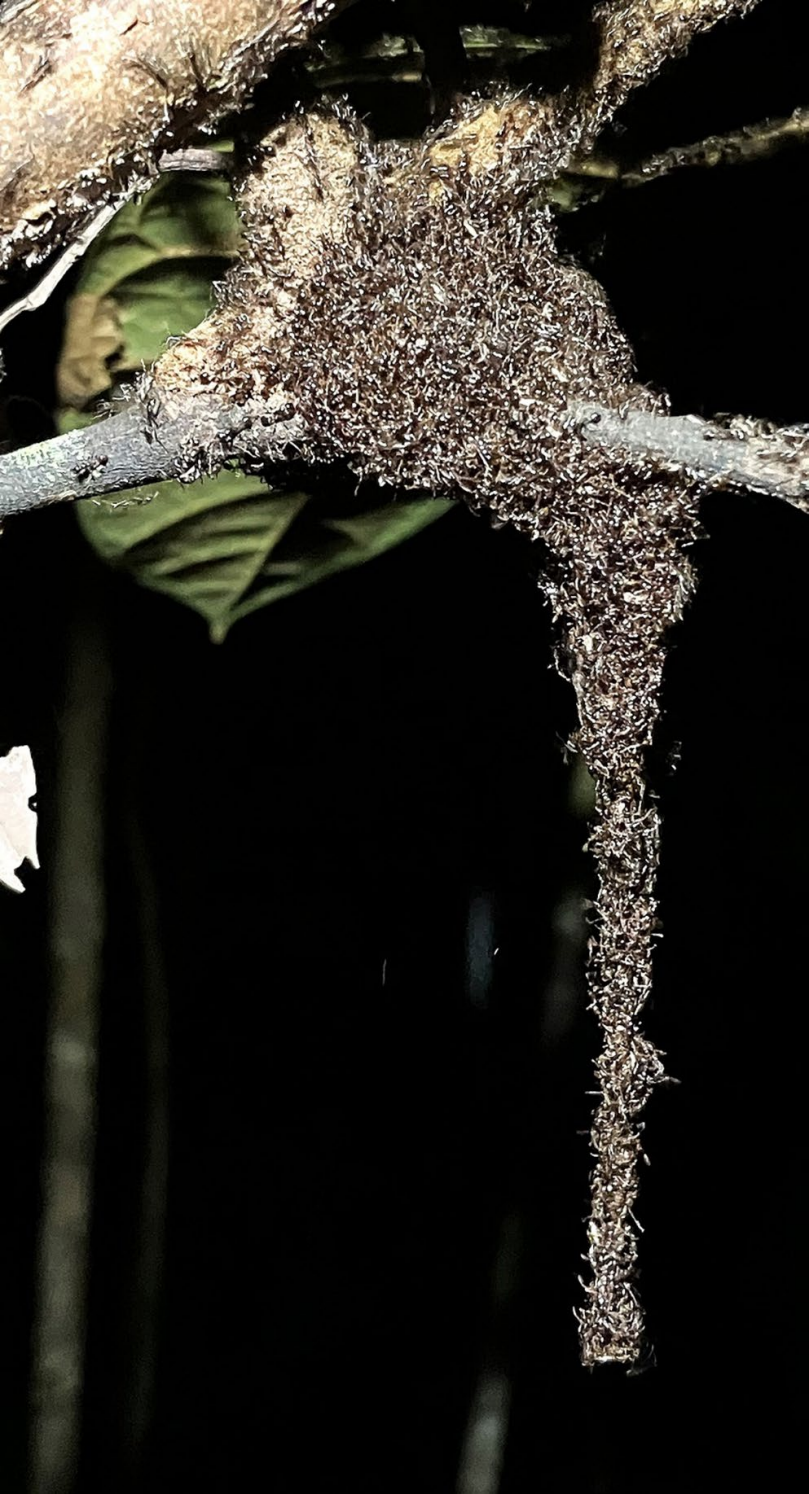
A self-assembled chain formation similar to a living bridge by *Aenictus glabrinotum*. The photo was taken on June 6, 2023, on the same day as the bridge observations in the same field locality (see Text  S1, Figure S4, and Video S2)

Living bridge formation in *Eciton* workers can emerge from simple behavioral rules. Individual ants track the rate of traffic passing over them and leave the bridge when tactile stimulation drops below a certain threshold (Powell and Franks 2007; Garnier et al. 2013; McCreery et al. 2022). Although our data cannot quantitatively test these behavioral rules, the observed traffic dynamics suggest that *A. glabrinotum* may employ a similar process. We recorded two spontaneous bridge collapses, and the second event coincided with declines in local traffic and ant density (Fig. 1). This should also be tested with multiple observations. Among self-assembly behaviors in social Hymenoptera, bivouac formation is the most widespread, providing a temporary shelter for colony members (Anderson et al. 2002; Carlesso and Reid 2023). Both Old World and New World army ants form bivouacs, including several *Aenictus* species (Gotwald 1995). We also observed a self-assembling bivouac of *A. glabrinotum* at our field site, including the hanging chain (Text S1, Figs. 2 and S4, Video S2). The underlying behavioral process governing bivouac and bridge formation might be shared in *Aenictus glabrinotum*.

In conclusion, we document the first evidence of living bridge formation in Old World army ants. Although this behavior is well known in *Eciton*, its evolutionary origin remains unclear. Unique foraging behavior in army ants, such as mass raiding, is suggested to have continuously evolved from group foraging (Chandra et al. 2021). Yet, behavioral information within the clade of Old World army ants of the subfamily Dorylinae remains scarce. Although based on a single observation, documenting behaviors of understudied but evolutionarily important species is a valuable natural history, particularly by generating hypotheses for future comparative studies. Our findings underscore the continuing importance of natural history observations in revealing behavioral diversity and convergence among social insects.

## Supplementary Information

Below is the link to the electronic supplementary material.

Supplementary Material 1

Supplementary Material 2

Supplementary Material 3

## Data Availability

Data and code for analysis are available at https://github.com/nobuaki-mzmt/AenictusLivingBridge, as well as Zenodo 10.5281/zenodo.18751735.
