# Supplementary material for: Independent evolution of a living bridge in the old world army ant lineage
Source: Naturwissenschaften. 2026 Mar 3;113(2):28. doi: 10.1007/s00114-026-02085-4 (PMC12956967; doi:10.1007/s00114-026-02085-4)
Supplement: Supplementary file 1 — Supplementary Material 1 [file 114_2026_2085_MOESM1_ESM.pdf]

Supplementary materials for

Independent evolution of a living bridge in the Old World army ant lineage

Nobuaki Mizumoto[1,2*], Kôichi Arimoto[3], Clement Het Kaliang[4], Taisuke Kanao[5]
1. Department of Entomology & Plant Pathology, Auburn University, Auburn, AL, 36849, USA
2. Okinawa Institute of Science & Technology Graduate University, Onna-son, Okinawa, 904-0495 Japan
3. Graduate School of Global Environmental Studies; Kyoto University; Kyoto; 606–8501 Japan
4. Sarawak Forestry Corporation, Kuching, Sarawak, Malaysia
5. Faculty of Science, Yamagata University, Yamagata, 990-8560, Japan
*: Correspondence: nzm0095@auburn.edu

ORCID: NM: 0000-0002-6731-8684; KA: 0000-0002-8703-8073, T.K.: 0000-0002-7934-4011

This file includes

Supplementary Text S1
Table S1
Figure S1-3
The legend for Video S1 and S2

Video S1. Observations of the bridge formation by *Aenictus glabrinotum*.
Video S2. Observations of the bivouac formation by *Aenictus glabrinotum*.

Supplementary Text S1

Species-level identification

In this study, we observed behaviors of *Aenictus* ants twice on June 6, 2023. We found ants that formed a self-assembly hanging chain at 9:13 pm (Fig. 2), and one hour later, we performed the experiment for the bridge formation (Fig. 1). The two locations we observed the ants were separated by about 100 m in straight-line distance, and apart from each other in about 250 m through a human-walkable trail. It is therefore highly likely that these two colonies were not the same.

We identified the species that showed a self-assembly hanging chain (Fig. 2) as *Aenictus glabrinotum* Jaitrong & Yamane, 2011 based on the external morphology of the voucher specimens (Fig. S4). However, we did not preserve the voucher specimens of the bridge (Fig. 1). Eighteen species in 11 species groups of *Aenictus* are known in the Lambir Hills National Park (Yamane et al. 2021). Identification of the *Aenictus* ants requires closer observation of the presence or absence of the typhlatta spot, the number of antennal segments, the density of hairs on the body surface, and the shapes of the head and thorax (Jaitrong and Yamane 2011). Many of the diagnostic features of the experimental ants could not be seen in the photos and videos taken in the field (Figs. 1 & S1, Video S1), but the thorax outline of the experimental ants could be confirmed (convex pronotum without horn-like protuberances, feebly convex propodeum, and rounded propodeal junction) in the videos. Only two species of the *Aenictus currax* group, *A. glabrinotum* and *A. pfeifferi* Zettel & Sorger, 2010, among the Lambir *Aenictus* species match these features. The other 16 species were excluded from consideration because they have a pronotum that is produced anteriorly into horn-like protuberances; a straight dorsal outline of the propodeum; an inflated propodeum; or a somewhat angular propodeal junction (Terayama and Yamane 1989; Yamane and Hashimoto 1999; Jaitrong et al. 2010; Jaitrong and Yamane 2011; Jaitrong et al. 2012; Jaitrong and Hashimoto 2012; Jaitrong and Yamane 2012; Jaitrong and Yamane 2013; Jaitrong 2015). If the experimental ant species is not an undiscovered species from the Lambir, it is likely one of the two known species.

Regarding the ecological information, four species in the Lambir, *A. cornutus* Forel, 1900, *A. glabrinotum*, *A. gracilis* Emery, 1893 and *A. laeviceps* (Smith, 1857) are known to forage on trees (Matsumoto et al. 2009; Jaitrong and Yamane 2011; Hashimoto and Yamane 2014). *Aenictus inflatus* Yamane & Hashimoto, 1999 is known to search for prey ant nests in the lower vegetation (Hashimoto and Yamane 2014).

Based on the information above, the experimental ant is most likely *A. glabrinotum*, though there is a slight possibility that this paper will provide new insights into the ecology of *A. pfeifferi*. In any case, *A. cornutus*, *A. glabrinotum*, *A. gracilis*, and *A. pfeifferi* belong to the *Aenictus currax* group. This indicates that the ability of arboreal use may be shared among species in this species group, as suggested by the results of the present study.

References

Hashimoto Y, Yamane S. 2014. Comparison of foraging habits between four sympatric army ant species of the genus *Aenictus* in Sarawak, Borneo. Asian Myrmecology. 6:1–10

Jaitrong W. 2015. A revision of the Thai species of the ant genus *Aenictus* Shuckard, 1840 (Hymenoptera: Formicidae: Dorylinae). The Thailand Natural History Museum Journal. 9:1–94

Jaitrong W, Hashimoto Y. 2012. Revision of the *Aenictus minutulus* species group (Hymenoptera: Formicidae: Aenictinae) from Southeast Asia. Zootaxa. 3426(1). https://doi.org/10.11646/zootaxa.3426.1.2

Jaitrong W, Yamane S. 2011. Synopsis of *Aenictus* species groups and revision of the *A. currax* and *A. laeviceps* groups in the eastern Oriental, Indo-Australian, and Australasian regions (Hymenoptera: Formicidae: Aenictinae). Zootaxa. 3128(1). https://doi.org/10.11646/zootaxa.3128.1.1

Jaitrong W, Yamane S. 2012. Review of the Southeast Asian species of the *Aenictus javanus* and *Aenictus philippinensis* species groups (Hymenoptera, Formicidae, Aenictinae). ZooKeys. (193):49–78. https://doi.org/10.3897/zookeys.193.2768

Jaitrong W, Yamane S, Tasen W. 2012. A sibling species of *Aenictus dentatus* Forel, 1911 (Hymenoptera: Formicidae) from continental Southeast Asia. Myrmecological News. https://doi.org/10.25849/MYRMECOL.NEWS_016:133

Jaitrong W, Yamane S, Wewatwitaya D. 2010. The army ant *Aenictus wroughtonii* (Hymenoptera: Formicidae: Aenictinae) and related species in the Oriental Region, with description of two new species. Japanese Journal of Systematic Entomology. 16:33–46

Jaitrong WJ, Yamane S. 2013. The *Aenictus ceylonicus* species group (Hymenoptera, Formicidae, Aenictinae) from Southeast Asia. Journal of Hymenoptera Research. 31:165–233. https://doi.org/10.3897/jhr.31.4274

Matsumoto T, Itioka T, Yamane S, Momose K. 2009. Traditional land use associated with swidden agriculture changes encounter rates of the top predator, the army ant, in Southeast Asian tropical rain forests. Biodiversity and Conservation. 18(12):3139–3151. https://doi.org/10.1007/s10531-009-9632-4

Terayama M, Yamane S. 1989. The army ant genus *Aenictus* (Hymenoptera, Formicidae) from Sumatra, with descriptions of three new species. Japanese Journal of Entomology. 57:597–603. https://doi.org/10.5281/zenodo.24868

Yamane S et al. 2021. A list of ants from Lambir Hills National Park and its vicinity, with their biological information: Part II. Subfamilies Leptanillinae, Proceratiinae, Amblyoponinae, Ponerinae, Dorylinae, Dolichoderinae, Ectatomminae and Formicinae. Contributions from the Biological Laboratory, Kyoto University. 31:87–157

Yamane S, Hashimoto Y. 1999. A remarkable new species of the army ant genus *Aenictus* (Hymenoptera, Formicidae) with a polymorphic worker caste. Tropics. 8(4):427–432

Table S1. The table for the results of GLM.
glm(traffic flow ~ Mean number of ants near the gap * Bridge formed)
Poisson, log link

	Estimate	SE	z	P (z score)
(Intercept)	3.06	0.10	31.75	< 0.001
Mean number of ants near the gap	0.05	0.01	7.59	< 0.001
Bridge formed	-0.53	0.21	-2.56	0.01
Interaction	0.02	0.01	2.17	0.03

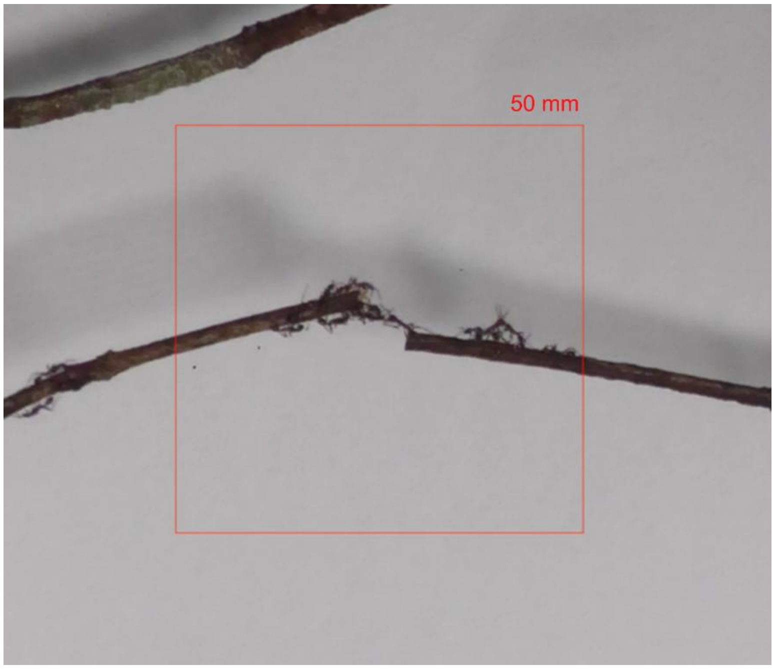

Figure S1. Observation area used in the BORIS behavioral observation. We defined the area as a square surrounding the gap in the twigs, measuring 50 x 50 mm.

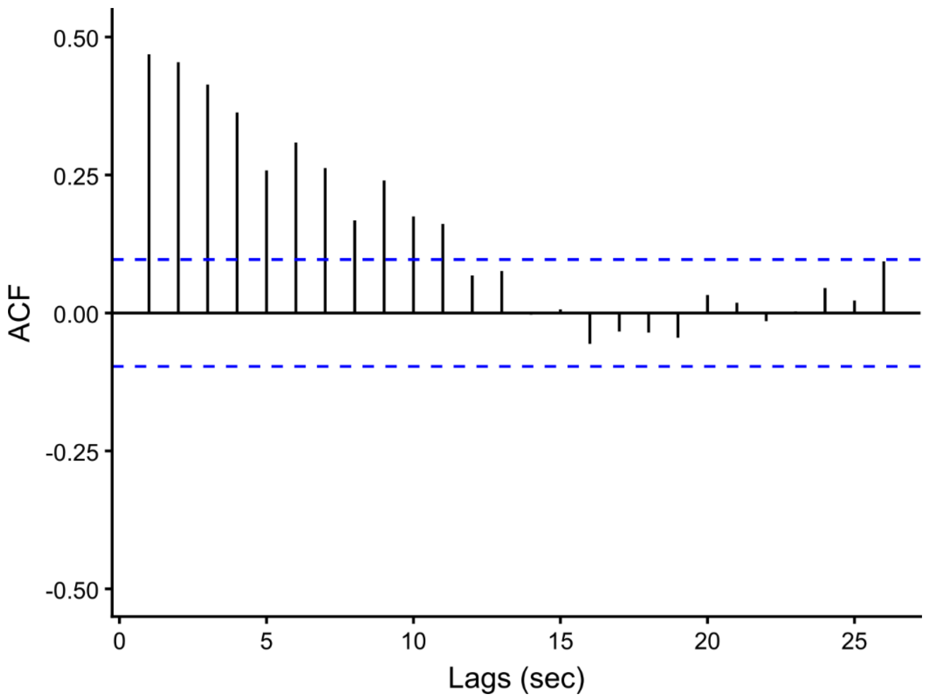

Figure S2. The autocorrelation function for the traffic flow. The traffic flow was obtained by summing the number of ants entering and leaving the area near the gap. Blue dashed lines indicate the 95% Confidence Interval, within which the signal cannot be distinguished from the noise.

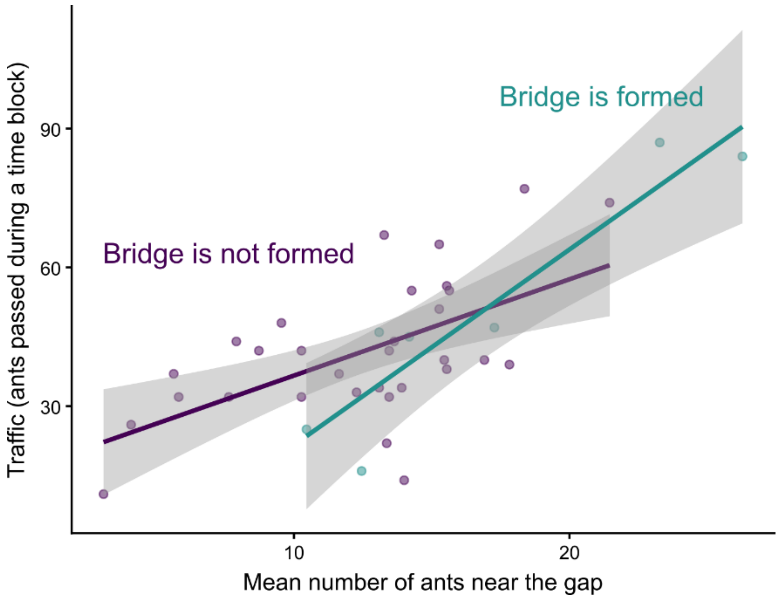

Figure S3. Relationship between the number of ants near the gap and traffic flow. Each data point represents the observation while 11 11-second time block (See also Figure S4).

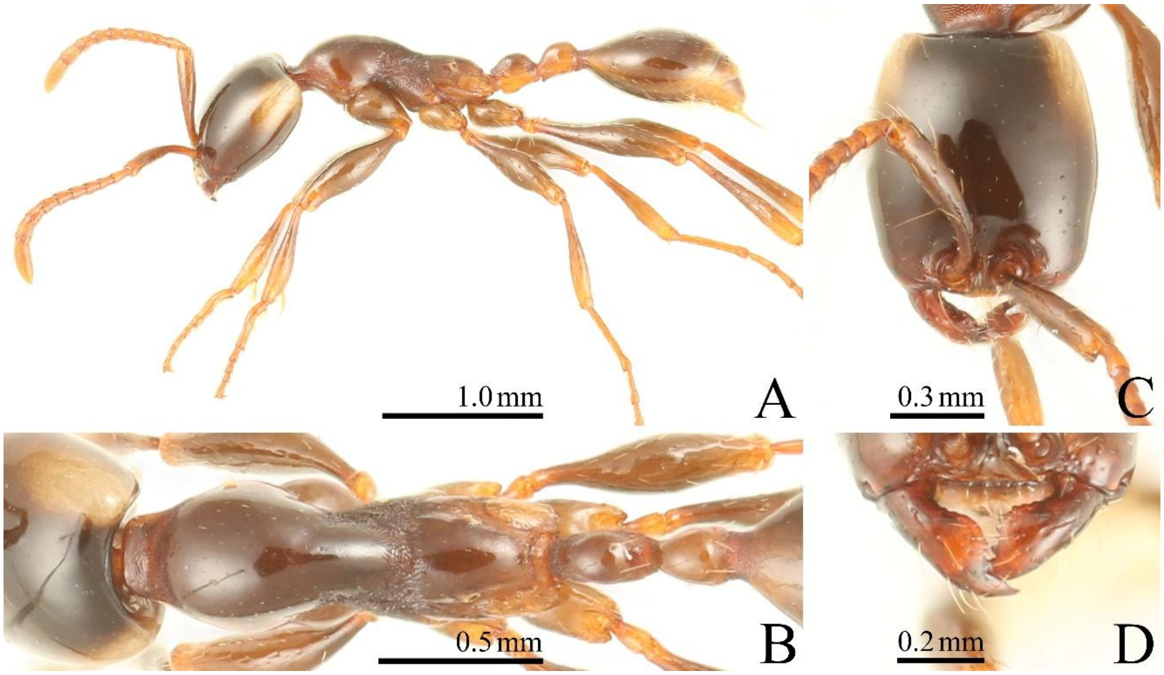

Figure S4. Worker of *Aenictus glabrinotum* Jaitrong & Yamane, 2011, forming a self-assembly hanging chain in figure 2. A. habitus in lateral view, B. thorax, petiole, and postpetiole in dorsal view. C. head in full-face view, D. anterior margin of head and mandibles in anteroventral view.
